# The Kinetics and Mechanism of the Reaction Between Serum Albumin and Auranofin (and its Isopropyl Analogue) *In Vitro*

**DOI:** 10.1155/MBD.1994.512

**Published:** 1994

**Authors:** Jacqueline R. Roberts, Jun Xiao, Brian Schliesman, David J. Parsons, C. Frank Shaw III

**Affiliations:** 1 Department of Chemistry University of Wisconsin-Milwaukee Milwaukee WI 53201-0413 USA; 2 Simulation Dynamics Inc. Milwaukee WI USA


					THE KINETICS AND MECHANISM OF THE REACTION
BETWEEN SERUM ALBUMIN AND AURANOFIN
(AND ITS ISOPROPYL ANALOGUE) IN VITRO
Jacqueline R. Roberts, Jun Xiao, Brian Schliesman
David J. Parsons2, and C. Frank Shaw III
Department of Chemistry, University of Wisconsin-Milwaukee, Milwaukee, Wl 53201-0413, USA
2 Simulation Dynamics Inc., Milwaukee, Wl, USA
The first detailed kinetic study of in vitro reactions between serum albumin and the second-
generation gold drug Auranofin [Et3PAuSATg triethylphosphine-(2,3,4,6-tetra-O-acetyl-l-8-D-
glucopytanosato-S-) gold(I)] and its tri-i-propylphosphine analogue, .iPr3PAuSATg, are reported. The
reactions were investigated using Penefsky spun columns and NMR saturation transfer kinetics.
Based on the Penefsky column data, the binding of the Et3PAuSATg to AIbSH (0.600 mM) was
complete when gold concentrations were limiting: 0.093, 0.151, and 0.225 mM. The reaction is
biphasic. The fast phase is defined by a first order rate constant, (k 2.94 _+ 0.24 x 10-2 sec"1)
and accounts for 95% of the Au(I) bound. This phase is first order with respect to albumin, and
zero order with respect to auranofin. A minor, slower step (k2 2.26 _+ 0.26 x 10-3 sec"1), which
accounts for only 5% of the reaction, is also first order with respect to albumin, and zero order with
respect to auranofin. For .iPr3PAuSATg, only the first step was observed, k 1.4 +_ 0.1 x 10-2 s"1
and is first order in albumin and independent of iPr3PAuSATg concentration. 31p.NMR saturation
transfer experiments utilizing the auranofin analogue, .iPr3PAuSATg, under equilibrium conditions
with excess .iPr3PAuSATg and ATgSH yielded second order rate constants for both the forward (1.0
x 102 M" sec"1) and the reverse (3.9 x 101 M"1 sec"1) directions. A multi-step mechanism
involving a conformationally altered albumin species to which gold binds was developed using the
steady-state approximation. The mechanism accounts for the different reaction orders observed
under the two set of conditions. A rate determining conformational change on the albumin governs
the reaction as monitored by the Penefsky columns. A second order reaction of R3PAuSATg at
Cys-34 is observed under the NMR conditions. These results are the first quantitative determination
of auranofin-albumin reaction kinetics and the first mechanistic study of the reaction.
A novel binding mechanism, association of auranofin in the pocket of albumin-disulfide
species can be detected by Penefsky and HummeI-Dreyer gel chromatographic techniques, but not
by conventional gel-exclusion chromatography. This complexalbumin-auranofin complex is weakly-
bound and readily dissociates.
512

				

